# High heart rate associated early repolarization causes J‐waves in both zebra finch and mouse

**DOI:** 10.14814/phy2.14775

**Published:** 2021-03-12

**Authors:** Joost A. Offerhaus, Peter C. Snelderwaard, Sila Algül, Jaeike W. Faber, Katharina Riebel, Bjarke Jensen, Bastiaan J. Boukens

**Affiliations:** ^1^ Department of Experimental Cardiology Academic Medical Center Amsterdam University Medical Center Amsterdam Netherlands; ^2^ Institute of Biology Leiden University Leiden The Netherlands; ^3^ Department of Medical Biology Academic Medical Center Amsterdam University Medical Center Amsterdam Netherlands

**Keywords:** early repolarization, J‐wave, mouse, zebra finch

## Abstract

High heart rates are a feature of small endothermic—or warm‐blooded—mammals and birds. In small mammals, the QT interval is short, and local ventricular recordings reveal early repolarization that coincides with the J‐wave on the ECG, a positive deflection following the QRS complex. Early repolarization contributes to short QT‐intervals thereby enabling brief cardiac cycles and high heart rates. We therefore hypothesized high hearts rates associate with early repolarization and J‐waves on the ECG of endothermic birds. We tested this hypothesis by comparing isolated hearts of zebra finches and mice and recorded pseudo‐ECGs and optical action potentials (zebra finch, *n* = 8; mouse, *n* = 8). In both species, heart rate exceeded 300 beats per min, and total ventricular activation was fast (QRS < 10 ms). Ventricular activation progressed from the left to the right ventricle in zebra finch, whereas it progressed from apex‐to‐base in mouse. In both species, the early repolarization front followed the activation front, causing a positive J‐wave in the pseudo‐ECG. Inhibition of early repolarization by 4‐aminopyridine reduced J‐wave amplitude in both species. Action potential duration was similar between ventricles in zebra finch, whereas in mouse the left ventricular action potential was longer. Accordingly, late repolarization had opposite directions in zebra finch (left‐right) and mouse (right‐left). This caused a similar direction for the zebra finch J‐wave and T‐wave, whereas in the mouse they were discordant. Our findings demonstrate that early repolarization and the associated J‐wave may have evolved by convergence in association with high heart rates.

## Graphical abstract

Zebra finches, a bird species with high heart rates, have a J‐wave on the ECG, which results from early repolarization, This supports the notion that J‐waves are a common phenomenon in endothermic animals with high heart rates.

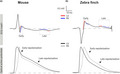

## 1 INTRODUCTION

High heart rates distinguish the hearts of mammals and birds from the hearts of ectothermic vertebrates (Hillman & Hedrick, 2015). The high heart rates are required to drive the great cardiac output needed to sustain the very energetically demanding state of endothermy (Boukens et al., 2019; Crossley et al., 2016). To achieve high heart rates, brief cardiac cycles are required.

The main determinant of the length of the cardiac cycle is the onset and duration of the repolarization phase of the ventricular action potential. During the repolarization phase, the cardiomyocyte is refractory or unexcitable, which is necessary to reset the intracellular calcium homeostasis. Regional differences in repolarization phase duration generate the T‐wave on the electrocardiogram (ECG). The QT‐interval is thereby an estimate for the duration of total ventricular repolarization. Across mammalian species, repolarization differences exist (Boukens et al., 2013; Detweiler, 2010; Opthof et al., 2017). For example, compared with humans, the action potential of mice is much shorter (Kaese & Verheule, 2012). In addition, the phase 1 repolarization is large and the plateau phase is absent. Consequently, the mouse ECG is without the isoelectric ST segment that in humans coincides with the plateau phase of the action potential. Instead, the murine early repolarization is visible on the ECG as a positive deflection directly following the QRS complex (Goldbarg et al., 1968). This so‐called J‐wave is seen in many species of rodents and was initially referred to as the rodent J‐wave (Gussak et al., 2000).

Not all rodent ECGs, however, exhibit a J‐wave and the Capybara, the largest rodent on earth exemplifies this (Szabuniewicz et al., 2010). Additionally, in other small non‐rodent mammals, such as shrews and bats, J‐wave‐like deflections follow the QRS complex (Currie, 2018; Nagel, 1986). What the animals of these groups share are high heart rates. To maintain the high heart rate, cardiomyocytes must be excitable upon arrival of the next activation front. For this, shortening of the action potential is crucial to overcome refractoriness and thereby ensuring excitability. This phenomenon is also reflected by the negative relationship between the QT interval and heart rate (Bazett, 1920).

If early repolarization is a mechanism to allow high heart rates, we also expect to find it in the hearts of other endothermic animals with high heart rates, such as various bird species. Numerous studies have looked at the electrocardiographic characteristics of different bird species (Table 1). Some bird species exhibit a non‐isoelectric ST segment, which could be due to early repolarization. It is known from human studies, however, that multiple phenomena can give rise to positive deflections after the QRS, such as hypothermia or late activation due to structural abnormalities (Boukens et al., 2020). Therefore, action potential recordings are required to confirm early repolarization as an underlying mechanism. Here, we electrically characterized excised hearts of the zebra finch (*Taeniopygia guttata*), a small bird with high resting heart rate (~600–700 bpm; Cooper & Goller, 2006), using pseudo‐ECGs (pECG) and optical action potentials and with comparisons to hearts of mice. Hearts of birds are strikingly similar to mammal hearts (Poelmann et al., 2014), although a large muscular right atrioventricular valve makes a conspicuous difference (Kroneman et al., 2019; Legler et al., 2019). The high similarity allowed us to submit the hearts of the two species to the same protocols. We hypothesized that the zebra finch pECG would exhibit a J‐wave that would be caused by early ventricular repolarization.

**TABLE 1:** Body mass (kg), heart rate (beats per minute), and ST segment (in lead II) in birds

Species	Body mass	Heart rate		Anesthesia	Clear isoelectric ST segment	Reference
Ostrich	~100		91	No	Present	Rezakhani et al. (2007)
Emu	41		69	Yes^a^	Present	Cushing et al. (2013)
Turkey	14		206	No	Present	Boulianne et al. (1992)
Andean condor	9.3		163	No	Present	Wiemeyer et al. (2013)
Whooper swan	9.2		85	No	Present	Machidad and Aohagi (2001)
Griffon vulture	6.4		160	No	Absent	Talavera et al. (2008)
Pekin duck	4		281	No	Absent	Cinar et al. (1996)
Green peafowl	4		258	No	Present	Hassanpour et al. (2011)
Muscovy duck	3		147	No	Absent	Hassanpour and Khadem (2013)
Golden eagle	2		347	No	Absent	Hassanpour et al. (2010)
Helmeted Guinea Fowl	1.3		338	No	Absent	Hassanpour et al., 2011)
Peregrine Falcon	0.80		268	No	Absent	Rodríguez et al. (2004)
Rook	0.55		340	No	Absent	Hassanpour et al. (2016)
Racing pigeon	0.52		211	No	Absent	Lumeij and Stokhof (1985)
Przevalski's partridge	0.51		317	No	Present	Liu and Li (2005)
Chukar partridge	0.42		317	No	Present	Uzun et al. (2004)
Pigeon (Spanish Pouter)	0.35		283	No	Absent	Lopez Murcia et al. (2005)
Teal	0.30		152	No	Present	Machidad and Aohagi (2001)
Japanese quail (male)	0.14		460	No	Absent	Szabuniewicz and McCrady (2010)
Laughing dove	0.13		357	No	Absent	Hassanpour et al. (2014)
Japanese quail (female)	0.12		320	No	Absent	Szabuniewicz and McCrady (2010)

^a^ Ketamine & Xylazine.

## 2 MATERIAL AND METHODS

### 2.1 Isolation of zebra finch hearts

Avian subjects were eight zebra finches (*Taeniopygia guttata*), four females and four males (mean age 14.7 months, ±6.0, mean body weight 17.7 g, ±1.0) that were raised in a breeding colony at Leiden University. After catching, the birds were immediately killed by cervical dislocation and the chest was opened. In two zebra finches, an *in situ* ECG was made after opening the chest and before excising the heart (Figure S1a). After excising the heart, a cannula was inserted in one of the three main branches of the aorta and fastened by a suture surrounding all three trunks. The arterial pole and coronary vessels were then flushed with ice‐cold cardioplegic solution (containing 110 mM NaCl, 1.2 mM CaCl_2_, 16 mM KCl, 16 mM MgCl_2_, 10 mM NaHCO_3_, and 9 mM glucose) to initiate diastolic arrest. The procedure from cervical dislocation to the flushing of the coronary vasculature took approximately 5 min. Once the coronary vessels were flushed, excess non‐cardiac tissue was trimmed off and the heart was placed in a reservoir of ice‐cold cardioplegic solution, a well‐known and standard method for heart transport for transplantation (Wahba et al., 2019), and measured within 1–4 h.

The use of postmortem material of animals culled as breeding surplus is not considered a procedure on itself in accordance with the Experiments on Animals Act (Wod, 2014). This is the applicable legislation in the Netherlands in accordance with the European guidelines (EU directive no. 2010/63/EU) regarding the protection of animals used for scientific purposes. Therefore, a license was not obtained for the procedure. All zebra finches were housed and cared for in accordance to these regulations and internal guidelines concerning care of the animals and licensing and skill of personnel. This also includes that advise was taken from the animal welfare body Leiden to minimize suffering for all animals at the facility (with or without a license).

### 2.2 Isolation of mouse hearts

Eight mice (*Mus musculus*) were used (FVB/NRj background, male, mean age 3.5 months, ±0.2) for the experiments. Mice were kept at the Amsterdam University Medical Center (AUMC), location AMC, animal breeding unit and had *ad libitum* access to Teklad 2916 chow (Envigo, Huntingdon, UK) and water. On the morning of the experiment, the mice were moved alive to the department of Experimental Cardiology. Mice were anesthetized by gradually increasing CO_2_ and were killed through cervical dislocation. Hearts were excised and the aorta cannulated as for zebra finches. All mouse experimental procedures were in accordance with governmental and institutional guidelines and were approved by the local animal ethics committee of the AUMC.

### 2.3 Optical mapping

The zebra finch and mouse hearts were mounted on a Langendorff perfusion setup and perfused at 37°C with Tyrode's solution (128 mM NaCl, 4.7 mM KCl, 1.45 mM CaCl_2_, 0.6 mM MgCl_2_, 27 mM NaHCO_3_, 0.4 mM NaH_2_PO_4_, and 11 mM glucose (pH maintained at 7.4 by equilibration with a mixture of 95% O_2_ and 5% CO_2_)), ﻿containing blebbistatin (20 μM, Bio‐Techne Ltd), an excitation–contraction uncoupler to prevent movement artifacts in the optical recordings. Hearts were submerged in HEPES buffered Tyrode's solution (140.2 mM NaCl, 5.4 mM KCl, 1.0 mM MgCl_2_, 1.8 mM CaCl_2_, 5.5 mM glucose, 5.0 mM HEPES). After a recovery period of ~10 min, the hearts received a bolus injection of di‐4‐ANEPPS (Molecular Probes), the fluorescent dye used for optical mapping. During 30 s, 0.4 mL of 20 μM di‐4 ANEPPS was injected. Pseudo‐electrograms were recorded by placing three electrodes at ±0.5 cm distance of the heart in the Einthoven configuration (Figure S2). Electrode R and L were placed alongside the right and left atrium, respectively, whereas electrode F was placed alongside the apex. In birds, the *in vivo* orientation of the heart is aligned with the spine and sternum such that the apex is pointing caudally and our electrode placement is therefore approximately the same as *in vivo* recorded avian body‐surface ECGs. Electrode R was used as negative input for both lead I and lead II. Recordings were made using Labchart amplifier (AD Instruments, Model 15T, sample frequency 1 kHz) and analyzed in LabChart Pro v8.1.13. The bipolar leads were measured (I = L − R, II = F − R, III = F − L). From these bipolar leads, augmented unipolar leads were calculated (aVR = R − (L + F)/2, aVL = L − (R + F)/2, and aVF = F − (L + R)/2), resulting in six‐lead pECGs. In birds, ECG deflections are known to have a low amplitude in lead I and we focused on lead II as is customary with avian ECGs (Whittow, 1999). Hearts were measured at 37°C (mice *n* = 8, zebra finch, *n* = 8) and during gradual increase to 42°C (zebra finch, *n* = 1). Activation and repolarization patterns were measured during sinus rhythm and atrial pacing at a basic cycle length of 120 ms (twice the diastolic stimulation threshold). In a subset of animals (mice *n* = 4, zebra finch *n* = 4), this was followed by treatment with 4‐aminopyridine (4‐AP, 0.5 mM, Sigma‐Aldrich). The protocol was repeated after a 10 min incubation period.

### 2.4 Analysis

Optical signals were analyzed using custom‐made software (Laughner et al., 2012) using Matlab2018. Onset of the QRS complex was taken as time zero for the beginning and duration of activation and repolarization. Local moment of activation was defined as the maximum positive d*V*/d*t* of the depolarization phase of the action potential. Repolarization times were determined at 20% and 80% of repolarization from an averaged (*n* = 10) optical action potential. In the mice, RT20 and RT80 analyses were possible in all 8 animals. However, due to movement artefacts we could only determine RT20 in 7 and RT80 in 4 zebra finches.

### 2.5 Statistics

Variables are presented as mean ± *SEM*. Activation times, repolarization times, heart rate, PR interval, QRS duration, and QT interval of zebra finch and mice were compared using unpaired t‐tests with *p* < 0.05 considered significant. RT20first, RT20last, RT80first, and RT80last in Figure 3b,c were each compared between ventricles and species using a two‐way analysis of variance (repeated factor: location (left or right ventricle) and fixed factor: species (mouse or zebra finch)). The effect of 4‐AP (Figure 6) was determined using a two‐way analysis of variance (repeated factor: treatment (before or after) and fixed factor: species (mouse or zebra finch)). In case of an interaction effect data were split and paired t‐test was performed within each species. All statistical analyses were made in SPSS (SPSS Statistics 25; IBM).

## 3 RESULTS

### 3.1 QRS complex, J‐wave, and T‐wave shape in the zebra finch ECG

**FIGURE 1:**
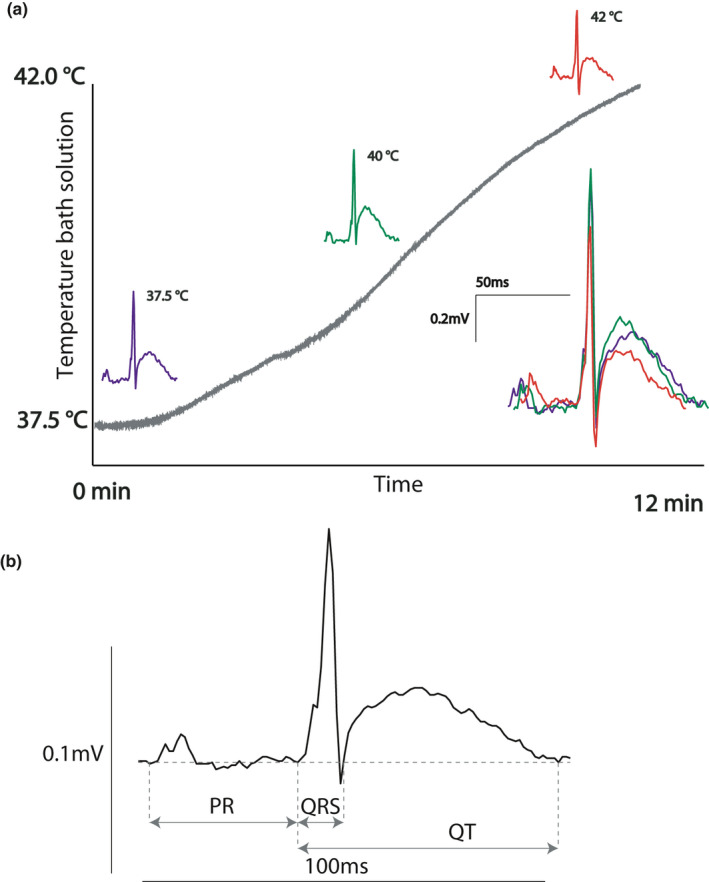
The zebra finch ECG. (a) The zebra finch pseudo‐ECG (pECG) measured at different temperatures had highly similar morphology, apart from shorted durations at higher temperatures. (b) Lead I of the zebra finch showing different pECG parameters

We recorded and compared pECG from Langendorff‐perfused hearts of zebra finch and mouse. All isolated hearts were spontaneously beating, showing a higher average heart rate in zebra finch than in mice (459.0 ± 36.3 vs. 336.3 ± 27.9 bpm, *p* = 0.018; Table 2). The body temperature in mice is 37°C (Reitman, 2018), whereas in zebra finches it is 39°C–42°C (Skold‐Chiriac et al., 2015). To be consistent between animals, all measurements were done at 37°C. To exclude a potential effect by hypothermia in the zebra finches, we first compared the zebra finch pECG at different temperatures (ranging from 37°C to 42°C). Although activation and repolarization occurred faster with higher temperatures, the ECG morphology was similar (Figure 1a). Table 2 presents the ECG parameters for the zebra finch compared with the mouse, which were measured as depicted in Figure 1b.

**TABLE 2:** ECG parameters for the zebra finch and Mouse

ECG parameters	Mouse (*n*)	Mean ± *SEM*	Zebra finch (*n*)	Mean ± *SEM*
HR (bpm)*	8	336.3 ± 27.9	8	459.0 ± 36.3
PR (ms)**	8	27.4 ± 0.7	8	49.8 ± 3.9
QRS (ms)	8	7.9 ± 0.4	8	8.6 ± 1.0
QT (ms)	8	70.7 ± 3.1	8	66.7 ± 3.1

* *p* < 0.05,

** *p* < 0.0001.

**FIGURE 2:**
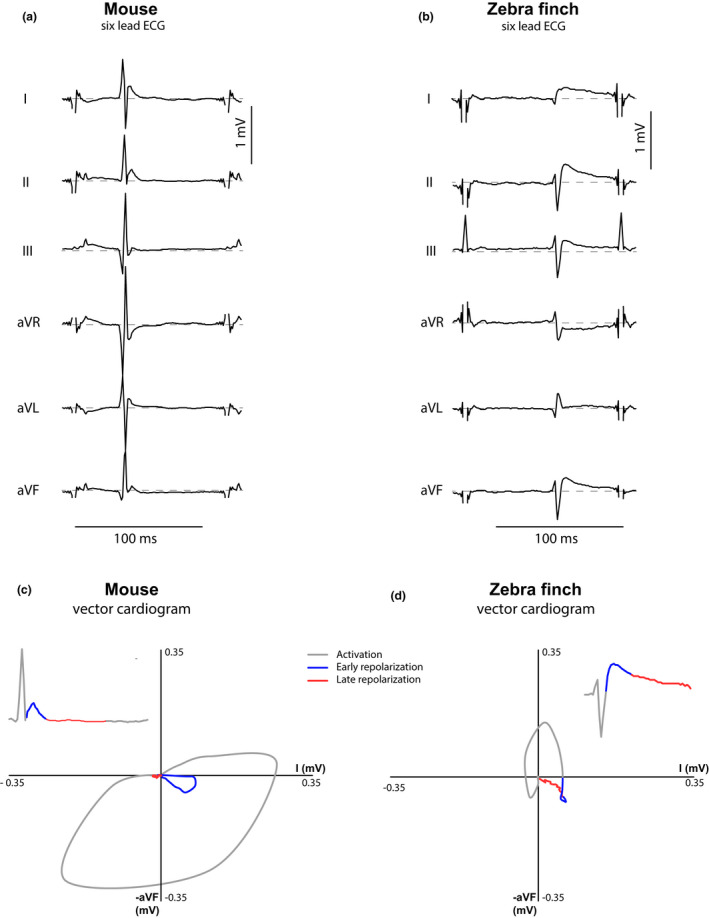
Six‐lead ECG and vector cardiograms for mouse and zebra finch: Contrary to mice, the J‐wave and T‐wave are concordant in the zebra finch. (a & b) Typical example of a six‐lead pseudo‐ECG in the mouse (a) and the zebra finch (b). Both animals show a positive deflection, the J‐wave, directly following the QRS. (c & d) Corresponding vector cardiograms based on leads I and aVF (inserts show Lead II traces, as species differences in the T‐wave are more pronounced in this lead). The direction of the early and late repolarizing electrical heart axes, characterized by the J‐wave and T‐wave respectively, are discordant in the mouse (c) and concordant in the zebra finch (d)

Figure 2a shows an example of the six‐lead pECG of the zebra finch (right) compared with the mouse pECG (left). For the zebra finch, the QRS complex in lead II was negative in all but one animal. In all mice, the QRS complex was positive in lead I. QRS duration did not differ between species (Table 2). In both species, the QRS complex was directly followed by a positive J‐wave. The J‐wave advanced into a T‐wave that intersected the isoelectric line on average at 66.7 ± 3.1 ms after the onset of the QRS complex in zebra finches and at 70.7 ± 3.1 ms in mice. The vector cardiograms in Figure 2c,d show typical examples of the electrical heart axis of the mouse and zebra finch during activation and repolarization. The J‐wave (colored blue) was positive in lead I in both species. By contrast, the T‐wave (colored red) was concordant with the J‐wave in all zebra finches and discordant in all mice (although with a low amplitude).

### 3.2 The pattern of ventricular activation differs between zebra finch and mouse

**FIGURE 3:**
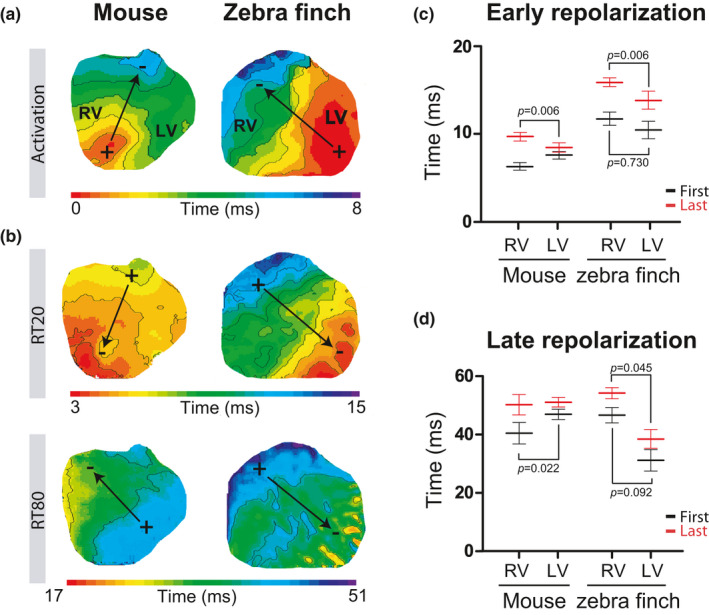
The early and late repolarization fronts follow the activation front in the zebra finch. (a & b). Typical activation (a) and repolarization (b) patterns in the mouse (left) and zebra finch (right). The black arrows indicate the electrical vector that is generated by the activation and repolarization front. Early repolarization was defined by 20% of total repolarization (RT20) and late repolarization by 80% of total repolarization (RT80). (c & d) The relationship between early (c) and late repolarization (d) in the right and left ventricle in mice (*n* = 8) and zebra finches (*n* = 7 for RT20 and *n* = 4 for RT80) is represented. Black and red mean and error bars represent start and end of repolarization, respectively. RV, right ventricle; LV, left ventricle. The *p*‐values below and above the bars refer to the comparison between the start and the end of repolarization, respectively, in the RV and LV

The timing of activation determines the onset of early repolarization, so we first established the activation sequence based on optical action potentials during atrial pacing. Figure 3a shows a typical example of the epicardial activation pattern at the ventral ventricular surface of a zebra finch and a mouse heart. In the zebra finch, the activation front propagated from the left ventricle (LV) to the right ventricle (RV) culminating with the activation of the right ventricular outflow tract (RVOT). In the mouse, in contrast, the activation front propagated from the apex to the base. Epicardial breakthrough of the activation front in the LV occurred earlier in zebra finch than in mouse (0.8 ± 0.3 vs. 2.0 ± 0.4 ms after the start of the QRS complex, *p* = 0.046). However, as with QRS duration, total activation time did not differ between the zebra finch and the mouse (6.0 ± 1.3 vs. 5.8 ± 0.3 ms, *p* = 0.876).

### 3.3 The pattern of ventricular early repolarization follows the activation pattern

To compare repolarization patterns in the zebra finches and mice, RT20 (early) and RT80 (late) patterns of repolarizations were analyzed. Figure S1b shows that in the zebra finch, RT20 and RT80 patterns did not change when heated from at 37°C to 42°C. In both zebra finches and mice, the pattern of early repolarization followed the activation pattern (Figure 3b). Both onset and final early repolarization occurred later in zebra finches than in mice (species effect *p* = 0.0001). In both species, early repolarization occurred first in the LV apex and last in the RVOT (location effect *p* = 0.006: 10.4 ± 1.0 ms vs. 15.9 ± 0.5 ms (zebra finch); 7.6 ± 0.5 ms vs. 9.8 ± 0.5 ms (mouse)). As a result, this electrical vector gives rise to the J‐wave with similar polarities in both animals in the six‐lead ECG (Figure 2). A vector caused by the repolarization front has an opposite direction to a vector generated by the activation front (as illustrated by the opposite direction of the arrows in Figure 3a,b). Accordingly, the polarity of the J‐wave was discordant with the polarity of the QRS complex in both species.

**FIGURE 4:**
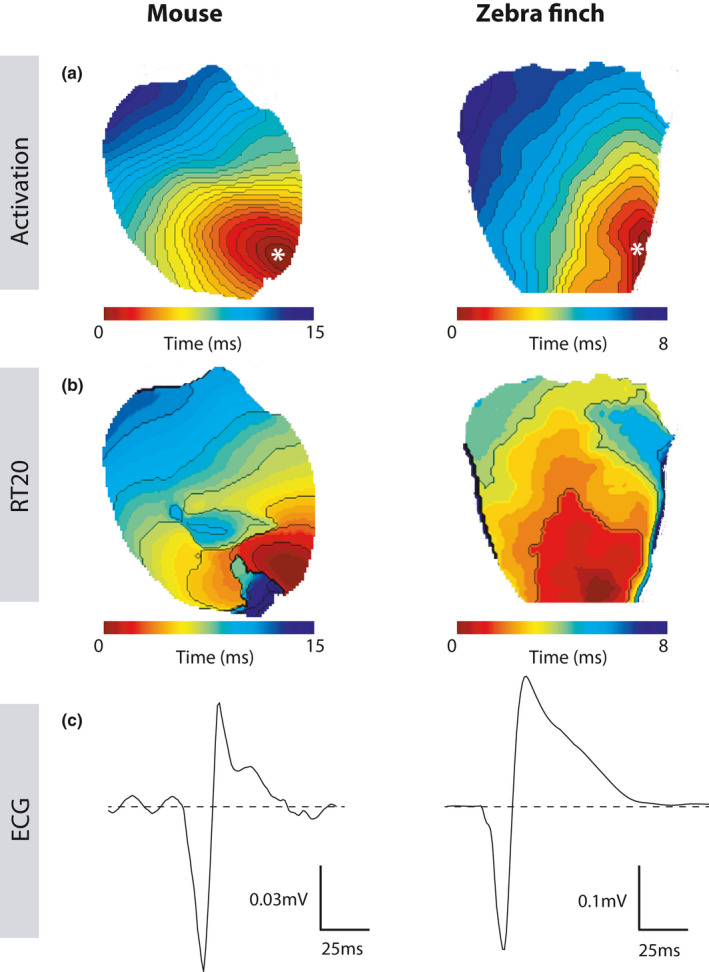
Apically paced mouse and zebrafinch hearts show similar activation and repolarization patterns resulting in the same J‐wave shape. (a & b) Apex paced hearts of mouse (left) and zebra finch (right) show similar activation patterns (a), resulting in similar morphology of the J‐wave seen on the accompanying pseudo‐ECGs (b). The white star indicates the place of stimulation

Since early repolarization followed the activation sequence in both species we tested whether ventricular pacing would give rise to similar QRS and J‐wave polarities. Figure 4 shows the activation and early repolarization pattern during left apical pacing in both a mouse and zebra finch heart. In both situations, the pECG showed a negative QRS complex followed by a positive J‐wave indicating that this morphology is the result of early repolarization, following the activation sequence.

### 3.4 Ventricular late repolarization is left‐right in zebra finch and right‐left in mouse

The pattern of late repolarization (RT80) differed markedly between mice and zebra finches (species × location *p* = 0.002; Figure 3b). In zebra finches, late repolarization started 31.0 ± 3.1 ms after onset of the QRS in the LV free wall and ended 54.5 ± 2.4 ms after onset of the QRS in the RV free wall. This pattern generated an electrical vector directed to the left side, leading to a positive T‐wave in lead II as observed in the pECG. In mice, however, late repolarization started 40.5 ± 3.7 ms after onset of the QRS in the RV free wall and ended 51.1 ± 1.6 ms after onset of the QRS in the LV free wall. This generated an electrical vector directed toward the right side, leading to a negative T‐wave in lead I as observed in the pECG.

### 3.5 The relation between the pECG and local ventricular optical potentials

**FIGURE 5:**
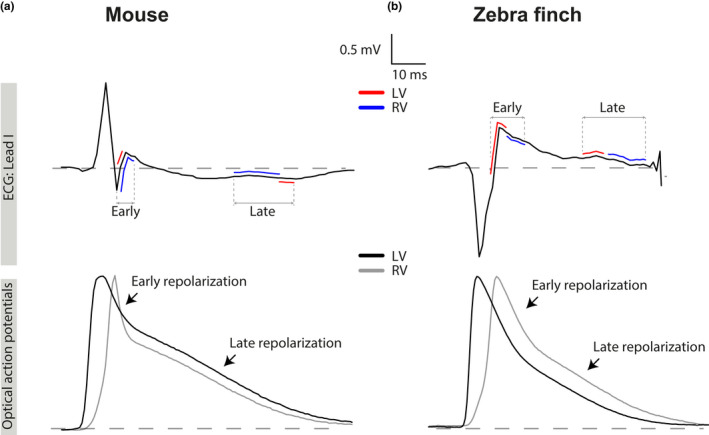
The relation between the J‐wave and T‐wave results from the order of early and late repolarization. (a & b) Lead I pseudo‐ECG (pECG) traces aligned with the simultaneously recorded optical action potentials from the LV (black) and RV (grey) in the mouse (a) and zebra finch (b). Early and late repolarization in LV (red) and RV (blue) are plotted on the pECG. The positive J‐wave is the result of an early repolarization front moving from LV to RV in both animals. In the zebra finch, late repolarization moves from LV to RV, resulting in a concordant T‐wave. In the mouse, however, the wave front moves from RV to LV resulting in a discordant T‐wave. LV, left ventricle; RV, right ventricle

Figure 5 shows the relation between local early and late repolarization and the J‐ and T‐wave in zebra finch and mouse. In the zebra finch, both early and late repolarization occurred later in the RV compared with the LV, causing a positive J‐wave and positive T‐wave in lead I. In mice, early repolarization ends later in the RV than LV, whereas late repolarization occurs earlier in the RV compared with the LV (Figure 5a). This latter phenomenon gives rise to a positive J‐wave and negative T‐wave in lead I.

### 3.6 Pharmacological reduction of early repolarization

**FIGURE 6:**
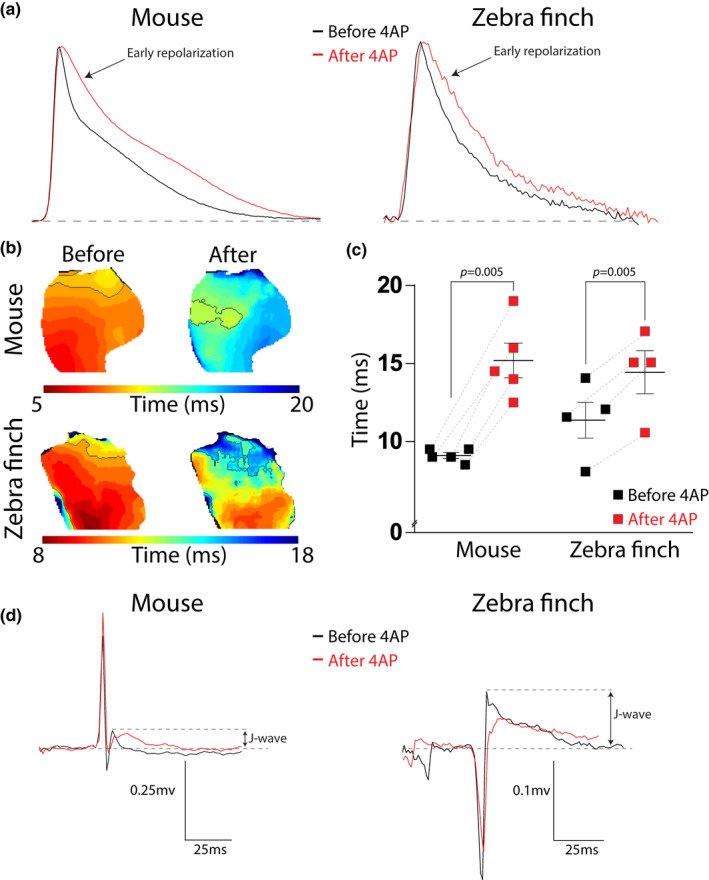
Blocking *I*_TO_ and *I*_Kur_ prolongs RT20 and attenuates the J‐wave in both mouse and zebra finch. (a–c) Typical examples of optical action potentials (a) and early repolarization patterns (b) of mouse (top) and zebra finch (bottom) exemplifying prolongation of RT20 (c) after administration of 4‐AP (in red). The*p* =0.005 refers to the main effect of the treatment. (d) Typical examples of the pseudo‐ECG before (black) and after (red) 4‐AP administration for mouse (left) and zebra finch (right). The attenuation of the J‐wave is measured as the percentage decrease in amplitude

We pharmacologically reduced phase 1 of the action potential by administering 4‐AP. The effect of 4‐AP on pECG parameters is presented in Table 3. 4‐AP did not affect total ventricular activation time (treatment *p* = 0.43), which was not different between species (species × treatment interaction *p* = 0.92). As expected, 4‐AP treatment resulted in a significant increase of RT20 compared with baseline (treatment *p* = 0.005) which was observed in both species (species × treatment interaction *p* = 0.219) (Figure 6a–c). In both mice and the zebra finches the RT20 prolongation—due to 4‐AP treatment—reduced J‐wave amplitude with respectively 34.7 ± 14.7% and 44.4 ± 18.0% (species × treatment interaction *p* = 0.92, treatment *p* = 0.0037; Figure 6d).

**TABLE 3:** Effect of 4‐aminopyridine

		Before		After (4‐AP)	
		Average	*SEM*	Average	*SEM*
RT20 (ms)	Mouse (*n* = 4)	9.1	0.2	15.2	1.1*
	Zebra finch (*n* = 4)	11.4	1.2	14.4	1.4*
QRS (ms)	Mouse (*n* = 4)	7.6	0.2	7.8	0.1
	Zebra finch (*n* = 4)	6.9	0.4	7.4	0.5
PR (ms)	Mouse (*n* = 4)	30.9	0.8	32.6	2.1*
	Zebra finch (*n* = 4)	40.6	2.9	53.9	8.1*
J Amplitude (mV)	Mouse (*n* = 4)	0.09	0.02	0.05	0.00*
	Zebra finch (*n* = 4)	0.05	0.02	0.03	0.01*

* *p* < 0.05.

## 4 DISCUSSION

Our data demonstrate the presence of J‐waves on the ECG of the zebra finch, a bird having a high heart rate. In both mouse and zebra finch, these J‐waves result from early repolarization that follows the activation front. Administration of 4‐AP slowed phase 1 repolarization and attenuated the J‐wave in both mouse and zebra finch. Our study supports the notion that J‐waves are not confined to rodents and further suggest that they are a common phenomenon in endothermic animals with high heart rates.

### 4.1 Activation and the QRS complex

Brief QRS duration is an example of convergent evolution between mammalian and avian hearts and is associated with higher heart rates than in reptiles which represent an approximate ancestral state of the mammalian and avian hearts (Boukens et al., 2019; Jensen & Christoffels, 2020). Hearts of mammals and birds also have a specialized ventricular conduction system and a compact wall composition both of which likely facilitates fast activation of the ventricles and brief QRS duration (Boukens et al., 2019; Davies & Francis, 1946). Compared to mammals the atrioventricular part of the conduction system is more developed in birds. There is a pronounced right atrioventricular ring bundle (Hoogaars et al., 2004; Prosheva et al., 2019), and the ventricular Purkinje system has a greater transmural penetrance (Davies & Francis, 1946; Gourdie et al., 1993). In mammals, the presence of the ventricular conduction system leads to two distinct breakthroughs of activation on the ventral surface of the left and right ventricle which is thought to reveal the presence of the left and right bundle branches of the His‐Purkinje system (Sedmera, 2011). Although we have observed these breakthrough before (Boukens et al., 2013), none of the mice measured in this study showed two breakthroughs. This could be due to the way of administering di‐4‐ANEPPS, which was via perfusion in this study and via superfusion in our previous study. When administering di‐4‐ANEPPS via perfusion, the contribution of deeper layered myocardium to the optical action potential measured at the epicardium is larger than using superfusion. We believe this may reduce the spatial resolution for detecting small regions of epicardial breakthrough. In birds, the ventricular conduction system is transmural (Davies & Francis, 1946), including chicken where large areas of epicardial breakthrough occur quickly after the onset of the QRS complex (Gourdie et al., 1993; Kharin et al., 2007). Epicardial activation in the zebra finch starts in the left ventricular free wall (near the apex) and propagates toward the base of RV. Although this pattern is different from that of mouse, it is specialised compared with the primitive base‐to‐apex pattern (Gregorovicova et al., 2018; Jensen et al., 2012). The zebra finch activation pattern is reflected by the negative QRS complex in lead aVF and lead II, which has long been recognized on the bird heart ECG (Whittow, 1999). Although we did not detect distinct early epicardial breakthroughs in the zebra finches, these have been reported for chicken, which belong to an older lineage of birds than zebra finches (Chuck et al., 2004; Kharin, 2004).

### 4.2 Early repolarization and the J‐wave

In both the zebra finch and mouse, the epicardial repolarization pattern resembled the activation pattern. Based on these patterns one expects that the J‐wave—resulting from the early repolarization pattern—is discordant with QRS complex (Opthof et al., 2016). However, this was only the case in the zebra finch and not in the mouse (Figure 2). We believe the explanation for this apparent paradox is that epicardial breakthrough in zebra finches coincides with the first moment of ventricular activation due to the presence of a transmural Purkinje system. In mice, however, the predominantly subendocardial Purkinje system allows for transmural activation occurring before the epicardial breakthrough (Miquerol et al., 2010), causing the breakthrough to occur much later after the onset of the QRS complex. This is illustrated by the positive QRS complex in aVF—indicating activation from base to apex—and the subepicardial activation pattern—which is from apex to base (Figure 3). Indeed, transmural activation occurring before the epicardial breakthrough in mice has been found to be an important component in determining QRS polarity in mouse (Liu et al., 2004). In mice, early repolarization in the subendocardium starts already when the parts of the ventricle have not been activated yet. Therefore, the J‐wave caused by this early repolarization is obscured by the QRS complex (Boukens et al., 2013). The J‐wave only becomes visible when the ventricular myocardium is fully activated and the QRS complex disappears. From that moment on, the J‐wave only represents the early repolarization difference between the base and the apex.

On the ECG, early repolarization causes a J‐wave in both mouse and zebra finch. In mice, early ventricular repolarization is caused by the currents *I*_Kur_ and *I*_to_ (Brouillette et al., 2004). Administering 4‐AP, a drug that reduces *I*_to_ and *I*_Kur_ (Xu et al., 1999), resulted in a prolongation of RT20 and attenuation of the J‐wave on the ECG in mice, a phenomenon also reported by others (Danik et al., 2002). Our data show that *I*_Kur_ and *I*_to_ are involved in early repolarization in zebra finches as well since administration of 4‐AP had similarly effects on RT20 and the J‐wave. A similar effect of 4‐AP on early repolarization has been show in isolated cardiomyocytes of Japanese quail (Filatova et al., 2021). This suggests that birds and mammals with high heart rates share a similar mechanism of early repolarization.

### 4.3 Late repolarization and the T‐wave

In lead I of mouse ECG, the J‐wave was followed by a negative T‐wave resulting from late repolarization starting in the right ventricle and ending in the left ventricle resembling what we have shown before (Boukens et al., 2013). In zebra finch, the pattern of late repolarization was opposite to that of mouse and started in the left ventricle and finished in the right ventricle. As a result, the J‐wave passed into a positive T‐wave. In both species activation differences between the left and right ventricle were small (±4 ms). Therefore, the gradients in late repolarization were mainly determined by differences in action potential duration, which in the zebra finch was much longer in the right ventricle than in the left ventricle (49.7 ± 2.3 vs. 28.0 ± 3.2 ms, respectively). In mammals, the action potential is substantially shorter in the right ventricle than in the left ventricle and the underlying mechanism is thought to involve embryonic, structural, and metabolic features (Molina et al., 2016). In both mammals and birds, the pulmonary arterial blood pressure is lower than the arterial systemic blood pressure (Seymour & Blaylock, 2000; Whittow, 1999), making it unlikely that differences in afterload between the ventricles explains the difference between species in action potential duration.

### 4.4 Translational perspective

J‐waves can occur in human as well with a prevalence varying between 5% and 19% (Offerhaus et al., 2020). In the majority of cases, these J‐waves are benign. However, J‐waves can also be the result of an underlying arrhythmogenic pathology, which is the case in the early repolarization syndrome and the Brugada syndrome, increasing the risk for lethal arrhythmias (Brugada & Brugada, 1992; Haissaguerre et al., 2008). It is thought that these so‐called J‐wave syndromes share early repolarization at a cellular level as a common mechanism underlying the J‐waves on the ECG and the occurrence of arrhythmias (Antzelevitch et al., 2016). However, recent studies have shown that delayed activation can lead to J‐waves as well (Boukens et al., 2020; Boukens, Opthof and Coronel, 2019; Hoogendijk et al., 2010). This may make the mechanism underlying J‐waves in mammals and birds with high heart rates different from that in human. Another occasion when J‐waves may occur is during hypothermia or after cardiac resuscitation, which are then referred to as Osborn waves (Jain et al., 1990; Osborn, 1953).

### 4.5 Evolutionary perspective

The higher heart rates of mammals and birds compared with reptiles are reflected in a QT duration which is approximately four‐fold shorter. Because the QT duration is the longest (ECG) interval of the cardiac cycle, the short QT duration appears to be a key adaptation to high heart rates (Boukens et al., 2019). The short QT intervals are the result of shorter ventricular action potentials which can be achieved by large phase 1 repolarization, as is the case in mice. Our data show a similar degree of large phase 1 repolarization in the ventricular myocardium of the zebra finch. This finding adds to the numerous features of convergent evolution between mammalian and avian hearts (Boukens et al., 2019; Kroneman et al., 2019). As of yet, there is no phylogenetic analysis of the occurrence of J‐waves in mammals or birds, and the number of evolutions of early repolarization is therefore not known. However, there are multiple species of bird in which the association between early repolarization and high heart rates could be tested. The ECGs of many birds, especially those with higher heart rates (>200 beats per minute), do not show a clear isoelectric ST segment and the S‐deflection often transitions directly into the T‐wave (Table 1). Although there are exceptions, for example the Turkey (Boulianne et al., 1992) or the Pekin duck (Cinar et al., 1996), we consider it likely that early repolarization contributes to the loss of the isoelectric ST segment and commonly occurs in birds with high heart rates.

### 4.6 Limitations

The isolated hearts we measured were disconnected from the autonomic nervous system which could have resulted in altered early and late repolarization patterns when compared with in‐vivo. Moreover, we used Di‐4 ANEPPS to record optical action potentials and blebbistatin to prevent motion artifacts in optical recordings. It has been suggested that both Di‐4 ANEPPS and blebbistatin affect local activation and repolarization patterns and thereby the shape and duration of the T‐waves (Kappadan et al., 2020; Larsen et al., 2012). Nevertheless, studies from our group (Boukens et al., 2013) and from others (Fedorov et al., 2007) have shown that action potential and ECG morphology is comparable in the absence and presence of Di‐4 ANEPPS and/or blebbistatin, suggesting that our findings can be translated to the in‐vivo situation.

## 5 CONCLUSION

We show that early repolarization in the zebra finch heart causes J‐waves on the ECG. This resembles the phenotype in mice and other small mammals with high heart rates. Our study supports the hypothesis that J‐waves coincide with higher heart rates.

## DISCLOSURE STATEMENT

The authors have no competing interest to declare.

## AUTHOR CONTRIBUTIONS

JAO, BJ, and BJB have performed the experiments and written the manuscript. PCS and SA assisted with experimental procedures and edited the manuscript. JWF and KR have critically read and edited the manuscript.

## Supporting information



Fig S1

Fig S2
