# Supplementary figures and images for: High heart rate associated early repolarization causes J‐waves in both zebra finch and mouse
Source: Physiol Rep. 2021 Mar 12;9(5):e14775. doi: 10.14814/phy2.14775 (PMC7953022; doi:10.14814/phy2.14775)

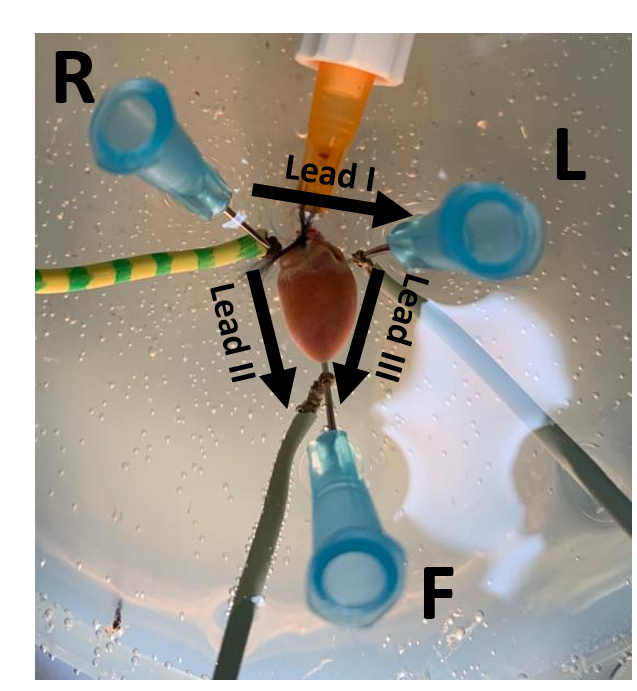

Supplement: Supplementary file 2 — Fig S2 [file PHY2-9-e14775-s001.png]
